# Factors associated with alcohol and/or drug use at sexual debut among sexually active university students: cross-sectional findings from Lebanon

**DOI:** 10.1186/1471-2458-14-671

**Published:** 2014-07-01

**Authors:** Lilian A Ghandour, Farah Mouhanna, Rola Yasmine, Faysal El Kak

**Affiliations:** 1 Department of Epidemiology and Population Health, American University of Beirut, Beirut, Lebanon; 2 Department of Health Promotion and Community Health, American University of Beirut, Beirut, Lebanon; 3 Department of Obstetrics and Gynecology, American University of Beirut Medical Center, Beirut, Lebanon

**Keywords:** Sexual debut, Youth, Alcohol, Drugs, Lebanon

## Abstract

**Background:**

Sexual activity accompanied by substance use can impair youth decision-making and enhance risk-taking behaviors. Less is known, however, about the sexual values, perceptions and subsequent sexual practices of youth whose sexual debut occurs while using alcohol/drugs.

**Methods:**

A cross-sectional anonymous online survey was conducted in April-August 2012 among undergraduate and graduate university students (aged 18 to 30) attending the 4th largest private university in Beirut. Pearson’s Chi-square and regression models were run using Stata/IC 10.0.

**Results:**

940 university students had engaged in oral, anal and/or vaginal sex, of whom 10% admitted to having had consumed alcohol or taken drugs at sexual debut, a behavior that was more common in the males, less religious, non-Arabs, students living alone or who had lived abroad. Students who used alcohol/drugs at sexual debut were twice as likely to have: their first oral and vaginal sex with an unfamiliar partner [odds ratio (OR) = 2.6, 95% confidence interval (CI): (1.6, 4.2) and OR = 2.1 (1.2, 3.5), respectively], controlling for sex, nationality, current relationship status, living abroad after the age of 12, and spirituality. Students who had sex the first time while using alcohol/drugs were three times as likely to report having had 11 or more subsequent sexual partners versus one or two [OR = 3.0 (1.5-6.0)]; and almost twice as likely to ever engage in something sexual they did not want to do [OR = 1.7 (1.1, 2.8)]. Perceived peer pressure to have sex by a certain age [OR = 1.8 (1.1, 2.9)], and perceived peer norms to consume alcohol/drugs before sex [OR = 4.8 (2.3, 9.9)] were also strong correlates of having sex for the first time while using alcohol and/or drugs.

**Conclusions:**

Findings stress the importance of sexuality education for youth, and the need to begin understanding the true interplay – beyond association - between youth sexual practices and substance use behaviors from a broader public health perspective.

## Background

Timing of sexual debut, occurring particularly during adolescence [1], reflects to a great deal youth personal sexual decision making [2], and the interplay of complex social and cultural factors [3]. The circumstances and behaviors surrounding sexual debut are important to understand as they have been shown to shape future sexual activity. Use of condoms during first-time sex among young adults in the United States (U.S.), for instance, has been linked to lesser risk of sexually transmitted infections (STIs) and a greater probability of using condoms during sexual activity years later, controlling for number of lifetime partners and frequency of sexual intercourse [4]. Similarly, in a general population survey in Slovenia, it was shown that sexually active men and women who used condoms during their first sexual intercourse were 11 and 2.5 times more likely to consistently use condoms during the month preceding the interview [5].

The context also matters. Early sexual experiences, especially the first, are important in sexual development as they influence one’s sexual script development [6]. First-time sex in a negative context such as, having sex coercively or with a sex-worker or under the influence of alcohol or drugs, has been shown to significantly increase odds of sexual dysfunctions, more frequent sex guilt, poorer self-reported physical health, more reported lifetime STIs, and less life satisfaction [6].

Alcohol and/or drug use at sexual debut, per se, has been linked to higher odds of regretting first time sex [7] and experiencing feelings of guilt particularly among women [8]. Additionally, having been drunk and/or stoned during first heterosexual intercourse (FHI) was associated with not only regretting first sex but also difficulty in expressing affection with a partner, even after adjusting for a group of structural characteristics, immediate social influences and other circumstances of first sexual intercourse including age, planning, contraception use and relationship to partner, all at sexual debut [7].

Although alcohol and/or drug use at sexual debut can be incidental, deliberate use to achieve sexual objectives has been reported. Alcohol is mostly used to facilitate a sexual encounter while cocaine and cannabis help enhance sensations and arousal [9]. Irrespective of the underlying reasons for alcohol/drug use at sexual debut, the latter is likely to result in less informed decisions [10-13] and more risk-taking by reducing consideration for contraception and STI prevention [14].

First-time sex while using alcohol and/or drugs can also raise questions about the choice of sexual partner (e.g., level of intimacy) and whether consensual sex took place. Previous studies have linked alcohol/drugs use at sexual debut to lack of discussion of risk-related topics with partner prior to sexual debut, and a reported low degree of intimacy with partner [5,15].

The plethora of the literature has examined the association between early sexual initiation and later substance use problems [16-18] and fewer have examined the health implications of sexual debut under the influence of alcohol and/or drugs [5-7,15]. Yet, and to our knowledge, no study has yet tried to understand other important differences in sexual values, attitudes or subsequent sexual practices between young people who choose to or happen to engage in alcohol/drug use at sexual debut versus not.

Lebanon, a small Arab country in the Middle East, constitutes an interesting context for investigating youth sexual choices and their impact on health. First, the topics of sexuality and sexual health remain socially tabooed, particularly in unmarried youth [19]. Premarital sex is socially tabooed, and women in particular are expected to remain virgins until married [20]. Readily available and valid informational resources are lacking, aggravated by the absence of a national school-based sexual health education program. Moreover, youth-friendly sexual health clinics are still short of meeting youth needs. Research focused on youth sexual practices and sexual health also scarce. In a recent study, vaginal sexual intercourse was reported by 7% of female and 47.7% of male university youth [21], estimates that we hypothesize are probably underestimated (particularly in females) given the increased number of young women seeking hymen repair following premarital sexual intercourse, reflecting probable changes in the social and cultural values of youth in the country [22].

This study from Lebanon expands on the extant literature on sexual debut and aims at investigating the following research question: how do sexually active youth who reported the use of alcohol and/or illegal drugs during their first penetrative sexual experience (versus youth who reported no alcohol or drug use at sex debut) vary with regards to their subsequent sexual practices, as well as sexual perceptions/values, and patterns of communication on sexual matters with parents, partners and peers?

## Methods

### Sample and data collection

This study was based on a cross-sectional self-completed anonymous online survey in English; of the total 7841 undergraduate and graduate students registered in the selected large private university during Spring 2013, a total of 2553 agreed to voluntarily participate in the survey (response rate 33%); yet only 2180 students met the eligibility criteria for age (18–30 years). Among the 2183 eligible participants, a total of 1839 provided a response (i.e. no, yes, rather not say) to all 3 questions inquiring about penetrative sexual activity: *“Have you ever had oral sex?”, “Have you ever had vaginal sex?”* and *“Have you ever had anal sex?”*; 943 students were identified as ‘ever having had penetrative sexual activity’ (i.e. ever engaged in oral, anal and/or vaginal sex), constituting the study sample for this analyses. Of the 943, 104 reported using alcohol and/or drugs at sexual debut (versus 829 who did not).

Sexually active students were aged about 21 years, comprised of a slightly higher percentage of males (55%), and were predominantly either Lebanese only or held dual citizenship (84%). They were mostly undergraduates (74%) and enrolled a non-health related faculty. They were equally split between being single and in a relationship, but the majority (76%) perceived themselves as financially dependent or mostly dependent. The vast majority of the sample was Lebanese (having one or dual citizenship, 84.4%), 9% were foreign Arab and 6.6% were foreign non-Arab. About 63% lived with their parents/guardians or partners/spouse, 23 independently in an apartment (alone or with roommate) and 14% lived in dorms or with siblings. Sexually active students came from a variety of high schools: 8% public schools, 33% private religious, and 59% private non-religious. Their reported levels of religiosity/spirituality were also heterogeneous: 25% religious/spiritual or very religious/spiritual, 35% somewhat religious/spiritual, and 40% not religious/spiritual or not at all religious/spiritual. Half the sample had lived in Lebanon all their life and 33% reported living abroad after the age of 12 for an uninterrupted period of six months or more.

### Ethical considerations

This study was granted approval by the University’s Institutional Review Board (IRB), and Human Research Protection Program (HRPP) affiliated with the American University of Beirut (AUB), protocol number: FHS.LG.04. *LimeSurvey’s* non-shareable secure survey hyperlink was used and the investigators were blinded to the students’ emails (i.e. the responses were non-traceable). Participating students agreed to participate by clicking “*Yes, I voluntarily agree to participate in this study research”* at the end of the online consent form; they were also given the right to refrain from answering particular questions and/or clear and exit the survey whenever they wanted.

### Instrument and selected measures

The self-administered questionnaire used was developed in English over a 5 months period after careful revision of six published sexual health surveys [23-28]. Culturally relevant questions reflecting the local context and norms surrounding sexual and reproductive health were also included (e.g., questions on hymen protection, temporary marriages, gender roles, pre-marital sexual relations, sexual pleasure). The questionnaire was designed to ease the participants into gradually answering more intimate and sensitive questions. A terminology box explained some sex-related terms (i.e. oral, anal, and vaginal sex, sexual intercourse, cybersex, masturbation, and outer-course). The questionnaire was pilot-tested among 14 recently graduated research assistants, and after each section feedback was solicited regarding the clarity, appropriateness, flow and sequence of the questions.

Selected measures for this current paper include the main variable of interest, *sexual debut while using drugs/alcohol*, which was assessed as follows: *“The first time you had any sexual intercourse (oral, vaginal or anal sex) had you been drinking or using drugs?”*

*Socio-demographics* included (listed in Table 1): sex, nationality, type of high school (public, private religious or private non-religious), educational level (undergraduate versus graduate or post-graduate), faculty (health-related as in health sciences or medicine versus non-health related), current relationship status (single including separated, divorced or widowed, or in a relationship including engaged, married or with a steady partner), years lived in Lebanon (not all my life vs. all my life), living abroad after the age of 12 (no, yes in one place, or yes in multiple places), and financials (dependent or independent). Students were also asked about their extent of spirituality/religiosity (“How religious or spiritual are you?”), responses ranging from very spiritual/religious to not spiritual/religious at all.

**Table 1:** Sociodemographic correlates of sexual debut while using alcohol or drugs among 943 sexually active university students

**Demographic**		**Sexual debut with usage of alcohol and drugs**				**Odds ratio**	
		**No**		**Yes**			
		**n**	**%**	**n**	**%**	**OR (95% CI)**	**P-value**
**Sex**	Female	397	93.2	29	6.8	1.00	-
	Male	432	85.2	75	14.8	**2.37 (1.51-3.73)**	**0.0001**
**Nationality**	Lebanese	471	89.9	53	10.1	1.00	-
	Dual citizen	237	90.5	25	9.5	0.93 (0.56, 1.55)	0.80
	Arab foreign	74	87.1	11	12.9	1.32 (0.65, 2.64)	0.42
	Non-Arab foreign	47	75.8	15	24.2	**2.83 (1.48, 5.41)**	**0.002**
**Type of High school**	Public	65	82.3	14	17.7	1.00	-
	Private, religious	284	92.5	23	7.5	**0.37 (0.18, 0.77)**	**0.01**
	Private, non-religious	475	87.8	66	12.2	0.64 (0.34, 1.21)	0.17
**Educational Level**	Undergraduate	604	88.1	82	11.9	1.00	-
	Graduate & Post-grad	222	91.4	21	8.6	0.69 (0.42, 1.15)	0.16
**Faculty**^**1**^	Health-Related	718	88.3	95	11.7	1.00	-
	Non-health-related	106	92.2	9	7.8	0.64 (0.31, 1.31)	0.23
**Current relationship status**^**2**^	Single	399	85.8	66	14.2	1.00	-
	In a relationship	430	91.9	38	8.1	**0.53 (0.35, 0.81)**	**0.004**
**Living Situation**^**3**^	Not independent	538	91.5	50	8.5	1.00	-
	Semi independent	112	86.1	18	13.9	1.73 (0.97, 3.07)	0.06
	Independent	176	83.0	36	17.0	**2.2 (1.38, 3.48)**	**<0.001**
**Years lived in Lebanon**	Not all my life	400	86.4	63	13.6	1.00	-
	All my life	429	91.3	41	8.7	**0.61 (0.4, 0.92)**	**0.02**
**Lived abroad after age of 12**	No	572	91.5	53	8.5	1.00	-
	Yes in one place	208	86.0	34	14.0	**1.76 (1.11, 2.79)**	**0.02**
	Yes in multiple places	49	74.2	17	25.8	**3.74 (2.01, 6.95)**	**<0.001**
**Financials**	Dependent	623	87.9	86	12.1	1.00	-
	Independent	206	92.0	18	8.0	0.63 (0.37, 1.07)	0.092
**Spirituality or religiosity**	Very religious or spiritual to Religious or spiritual	226	96.6	8	3.4	1.00	-
	Somewhat	297	89.5	35	10.5	**3.33 (1.51, 7.31)**	**0.003**
	Not – not at all religious or spiritual	306	83.4	61	16.6	**5.63 (2.64, 12.00)**	**<0.001**

^1^Health-related= FHS and FM; Non-health-related faculties= FAFS, FAS, FEA, SN, and OSB.

^2^Single=Separated/Divorced/Widowed; in a relationship= being in a relationship/engaged/married.

^3^Not independent = living at home (with parents/guardians or with spouse/partners); Semi independent = living in an apartment with a roommate; independent = living at university dorms or in an apartment (alone).

Bolded p-values are statistically significant at critical α=0.05.

Students’ *sexual behaviors* at sexual debut and subsequently were also measured using 13 questions capturing reported familiarity with first sexual partner, level of consent at sexual debut, number of lifetime sexual partners, among other risky practices (listed in Table 2).

**Table 2:** Sexual behavioral correlates of sexual debut while using drugs or alcohol among 943 sexually active university students

**Behavioral correlates**		**Sexual debut while on drugs or alcohol**				**Odds ratio**		**Adjusted odds ratio by sex**		**Adjusted odds ratio**^**1**^	
		**No**		**Yes**							
		**n**	**%**	**n**	**%**	**OR (95% CI)**	**P-value**	**A-OR (95% CI)**	**P-value**	**A-OR (95% CI)**	**P-value**
**Engagement in first oral sex with**	Familiar partner^2^	617	80.2	54	56.3	1.00	-	1.00	-	1.00	-
	Unfamiliar partner	152	19.7	42	43.7	**3.15 (2.03, 4.90)**	**<0.0001**	**2.67 (1.66-4.28)**	**<0.0001**	**2.56 (1.57-4.16)**	**<0.0001**
**Engagement in first anal sex with**	Familiar partner	201	79.1	16	72.7	1.00	-	1.00	-	1.00	-
	Unfamiliar partner	53	20.9	6	27.3	1.42 (0.53, 3.81)	0.48	1.18 (0.41, 3.37)	0.72	1.27 (0.44, 3.71)	0.66
**Engagement in first vaginal sex with**	Familiar partner	383	75.2	46	53.5	1.00	-	1.00	-	1.00	-
	Unfamiliar partner	126	24.8	40	46.5	**2.64 (1.65, 4.22)**	**<0.0001**	**2.10 (1.24, 3.51)**	**<0.0001**	**2.05 (1.2, 3.49)**	**0.01**
**Level of consent at sexual debut**	Willing	515	83.9	72	81.8	1.00	-	1.00	-	1.00	-
	Not willing^3^	19	3.09	3	3.4	1.13 (0.33, 3.91)	0.85	1.34 (0.38, 4.72)	0.65	1.96 (0.51, 7.5)	0.33
	Persuaded	59	9.61	6	6.8	0.73 (0.30, 1.75)	0.48	0.85 (0.35, 2.05)	0.71	0.98 (0.39, 2.45)	0.97
	Expected to	21	3.42	7	8.0	2.38 (0.98, 5.81)	0.06	2.18 (0.89, 5.36)	0.09	2.42 (0.94, 6.2)	0.07
**Paid for sex**	No	739	89.6	87	84.5	1.00	-	1.00	-	1.00	-
	Yes	86	10.4	16	15.5	1.58 (0.88, 2.81)	0.12	1.11 (0.61, 2.03)	0.72	1.10 (0.59, 2.04)	0.75
**Condom usage**	Always have	136	22.0	28	30.8	1.00	-	1.00	-	1.00	-
	Not always	481	78.0	63	69.2	0.64 (0.39, 1.03)	0.07	0.72 (0.44, 1.17)	0.18	0.63 (0.37, 1.05)	0.08
**Condom use at most recent sex**	Yes	345	56	54	59.3	1.00	-	1.00	-	1.00	-
	No	271	44	37	40.7	0.87 (0.56, 1.36)	0.55	1.03 (0.65, 1.62)	0.91	0.99 (0.61, 1.58)	0.95
**Number of lifetime sexual partners**	1-2	302	49.8	25	28.7	1.00	-	1.00	-	1.00	-
	3-5	181	29.9	36	41.4	**2.40 (1.39, 4.13)**	**0.002**	**2.14 (1.23, 3.71)**	**0.01**	**1.85 (1.05, 3.25)**	**0.03**
	6-10	68	11.2	11	12.7	**1.95 (0.91, 4.16)**	**0.08**	**1.70 (0.79, 3.67)**	**0.17**	1.31 (0.60, 2.89)	0.49
	11 +	55	9.1	15	17.2	**3.29 (1.63, 6.64)**	**<0.001**	**2.97 (1.45, 5.99)**	**0.003**	**2.06 (0.98, 4.29)**	**0.05**
**Ever experienced an unplanned pregnancy**	No	790	95.5	100	96.1	1.00	-	1.00	-	1.00	-
	Yes	37	4.5	4	3.9	0.85 (0.29, 2.44)	0.76	1.02 (0.35, 2.97)	0.96	0.95 (0.31, 2.88)	0.93
**Ever done something sexual didn’t want to do**	No	585	71.2	61	59.2	1.00	-	1.00	-	1.00	-
	Yes	203	24.7	33	32.0	**1.56 (0.99, 2.45)**	**0.05**	**1.92 (1.21, 3.01)**	**<0.001**	**1.74 (1.07, 2.82)**	**0.02**
	Don’t know/remember	34	4.1	9	8.8	**2.54 (1.16, 5.54)**	**0.01**	**2.58 (1.17, 5.67)**	**0.01**	**2.32 (1.02, 5.23)**	**0.04**
**Ever been in relationship moving too fast physically**	No	462	56.4	68	65.4	1.00	-	1.00	-	1.00	-
	Yes	357	43.6	36	34.6	0.68 (0.44, 1.04)	0.08	0.76 (0.49, 1.18)	0.23	0.86 (0.55, 1.34)	0.52
**Ever subjected to sexual abuse and/or harassment**	No	650	81.7	86	84.3	1.00	-	1.00	-	1.00	-
	Yes	146	18.3	16	15.7	0.83 (0.47, 1.45)	0.51	1.08 (0.60, 1.93)	0.79	0.91 (0.49, 1.65)	0.74
**Extent worried about doing more sexually than intended due to alc/drugs**	Not worried at all	422	57.2	46	46	1.00	-	1.00	-	1.00	-
	Not too worried	202	27.4	37	37	**1.68 (1.05, 2.67)**	**0.03**	1.59 (0.99, 2.53)	0.06	1.27 (0.78, 2.05)	0.32
	Somewhat worried	74	10	10	10	1.24 (0.59, 2.56)	0.56	1.36 (0.65, 2.82)	0.41	1.09 (0.51, 2.29)	0.83
	Very worried	40	5.4	7	7	1.6 (0.68, 3.78)	0.28	1.88 (0.78, 4.46)	0.15	1.63 (0.67, 3.96)	0.28

^1^Controlling for sex, nationality, current relationship status, living abroad after the age of 12, and spirituality.

^2^Familiar partner = serious partner, friend, or marital spouse. Unfamiliar partner = random partner or sex worker.

^3^Not willing = tricked, forced or coerced (received money, food, clothing, gifts).

Bolded p-values are statistically significant at critical α=0.05.

*Patterns of communication* on sexual matters with most recent partner, close friends as well as with parents/guardians were assessed via 4 questions. Finally, *sexual values/perceptions* were assessed using 8 statements measured on a Likert scale (strongly agree-strongly disagree); 3 categories were recreated to reflect agreement, indecisiveness or disagreement.

### Data analysis

Data analysis was conducted using Stata/IC 10.0. Frequencies were run for descriptive statistics and bivariate analysis was conducted using Pearson’s Chi-square. Three binary logistic regression models were conducted: a simple unadjusted model (model 1), one adjusted for sex only (model 2) and a model further adjusting for additional sociodemographic correlates that were statistically significantly associated with *sexual debut while using alcohol/drugs* at the bivariate level (model 3). The critical alpha level was set at 0.05.

## Results

### Sociodemographic correlates of sexual debut while using alcohol/drugs

Being a male and a non-Arab foreigner (vs. Lebanese) were associated with increased odds of consuming alcohol and/drugs at sexual debut; students of a non-Arab foreign nationality persistently had higher odds of using alcohol/drugs during first-time sex even after controlling for years lived in Lebanon and other sociodemographic characteristics (OR = 2.43; CI = [1.25; 4.73]; p-value = 0.009). Living in university dorms or in an apartment alone versus living at home with parents or spouse was also positively associated with alcohol and/or drug consumption during first-time sex (Table 1). Moreover, students who reported living in Lebanon their entire life were less likely to consume drugs/alcohol at sexual debut, but that was no longer statistically significant after controlling for nationality (OR = 0.66; CI = [0.41, 1.08]; p-value = 0.099); however, living abroad after the age of 12 for a period of 6 months or more was associated with higher odds of consuming drugs/alcohol at sexual debut (living in more than place with even a greater odds than in one place) (Table 1); the latter association held true even after adjusting for nationality [living abroad in one place (OR = 1.72; CI = [1.05, 2.81]; p-value = 0.031); more than one place (OR = 3.23; CI = [1.61; 6.50]; p-value = 0.001)]. Among the sexually active students, higher levels of reported religiosity were negatively associated with alcohol and/drug use at sexual debut (Table 1).

### Sexual practices linked to sexual debut while using alcohol/drugs

No observed differences in age at first sex (either oral or anal or vaginal) was found between students who reported using alcohol/drugs at sexual debut (mean age: 17.75 years) and those who did not (17.53 years, p-value = 0.426). Both sexually active groups were also equally likely to report ‘always using condoms’ during their lifetime, and using condoms during their ‘most recent sexual encounter’ (Table 2).

Still, and controlling for socio-demographics, sexually active students who reported consuming alcohol/drugs at sexual debut were more than twice as likely to report having engaged in their first oral and/or vaginal sex (but not anal sex) with an *unfamiliar partner* (i.e. random partner or sex worker) versus a familiar partner (i.e. serious partner, friend, or marital spouse) (Table 2). Worth noting that the percentage of students who reported engaging in first oral, anal and vaginal sex with an unfamiliar partner were 23%, 22% and 28%, respectively. Students who reported substance use at sexual debut were also more likely to report an increased number of lifetime sexual partners (controlling for sociodemographics) (Table 2); worth noting, 47% had reported 1–2 partners, 31% 3–5 partners, 12% 6–10 partners, and a sizeable 10% reported 11 partners or more. While level of consent was not statistically significantly related to substance use at sexual debut (with 84% having reported being ‘willing’), still students whose sex debut was under the influence were about twice as likely to have ‘*ever done something sexual that they did not want to do*’ (reported by about 26% of sexually active students) (Table 2).

### Patterns of communication

The majority (70%) of sexually active students reported talking to their current or last partner about what they feel comfortable doing sexually openly and freely; students who reported sexual debut while using alcohol and/or drugs were equally likely to communicate with their partners, compared to those who did not (Table 3).

**Table 3:** Association between sexual debut while using drugs or alcohol and patterns of sexual communication among 943 sexually active university students

**Communication**		**Sexual debut while on drugs or alcohol**				**Odds ratio**		**Adjusted odds ratio by sex**		**Adjusted odds ratio**	
		**No**		**Yes**							
		**n**	**%**	**n**	**%**	**OR (95% CI)**	**P-value**	**A-OR (95% CI)**	**P-value**	**A-OR (95% CI)**	**P-value**
**With partners**	No	54	6.8	7	7.1	1.00	-	1.00	-	1.00	-
	Somehow yes^1^	168	21.1	35	35.3	1.60 (0.67, 3.82)	0.28	1.64 (0.68, 3.9)	0.26	1.50 (0.63, 3.68)	0.34
	Yes openly and freely	574	72.1	57	57.6	0.77 (0.33, 1.76)	0.53	0.82 (0.35, 1.88)	0.63	0.72 (0.31, 1.70)	0.46
**With mother/female guardian**	No	511	62.2	68	65.4	1.00	-	1.00	-	1.00	-
	Undecided	75	9.1	8	7.7	0.80 (0.37, 1.73)	0.57	0.82 (0.37, 1.77)	0.61	0.79 (0.36, 1.72)	0.55
	Yes	236	28.7	28	26.9	0.89 (0.56, 1.42)	0.63	0.94 (0.59, 1.50)	0.80	0.91 (0.57, 1.47)	0.71
**With father/male guardian**	No	524	64.6	54	53.5	1.00	-	1.00	-	1.00	-
	Undecided	84	10.3	10	9.9	1.15 (0.56, 2.35)	0.69	0.90 (0.44, 1.86)	0.78	0.86 (0.41, 1.80)	0.69
	Yes	204	25.1	37	36.6	**1.75 (1.12, 2.75)**	**0.01**	1.22 (0.76, 1.99)	0.41	1.17 (0.72, 1.92)	0.51
**With friends**	No	107	13.5	4	3.8	1.00	-	1.00	-	1.00	-
	Yes	685	86.5	100	96.2	**3.90 (1.40, 10.83)**	**0.01**	**3.48 (1.25, 9.68)**	**0.02**	2.70 (0.96, 7.59)	0.06

^1^Somehow yes = using hints and cues. No = makes me or my partner uncomfortable.

Bolded p-values are statistically significant at critical α=0.05.

With regards to communication with parents, only 29% and 27% of the students felt they could talk to their mother/female guardian or father/male guardian, respectively; again, communication patterns with either parents/guardians did not differentiate students who reported use of drugs/alcohol at sexual debut from those who did not (Table 3).

The picture is different with ‘close friends’, whereby 88% of the sexually active student sample felt they could talk about sex with their close friends. "Communication with close friends" and "alcohol/drugs consumption at sexual debut" were strongly associated (OR = 3.9, Table 3), even after controlling for "communication with parents" (mom or dad) and all other sociodemographics [OR = 3.89; CI = (1.39; 10.89), p-value = 0.01].

### Sexual perceptions/values

Sexually active students who agreed that ‘*there was pressure to have sex by a certain age*’ and that ‘*It’s fun to experiment with strangers*’ were more than twice as likely to report sexual debut while using alcohol/drugs compared to those who disagreed; however, associations were no longer statistically significant when controlling for sociodemographics (Table 4). However, sexually active students who perceived that consumption of drugs or alcohol before sex took place ‘a lot’ (25% of the sample) versus ‘little or never’ were 4.76 times as likely to use alcohol/drugs themselves at sexual debut, controlling for socio-demographics (Table 4). Also adjusting for sociodemographics, sexually active students who agreed that ‘*only two people who trust each other should have sexual relations*’ were half as likely to have used alcohol/drugs during first-time sex (Table 4); students who admitted being ‘undecided’ were more than 2.5 times as likely to report sexual debut under the influence (Table 4).

**Table 4:** Association between sexual debut while using drugs or alcohol and youth sexual perceptions/values among 943 sexually active university students

**Perceptions**		**Sexual debut while on drugs or alcohol**				**Odds ratio**		**Adjusted odds ratio by sex**		**Adjusted odds ratio**^**1**^	
		**No**		**Yes**							
		**n**	**%**	**n**	**%**	**OR (95% CI)**	**P-value**	**A-OR (95% CI)**	**P-value**	**A-OR (95% CI)**	**P-value**
**Sex without a condom one in a while is a big deal**	Disagree	216	29.0	32	31.7	1.00	-	1.00	-	1.00	-
	Undecided	60	8.0	11	10.9	**1.24 (0.59, 2.60)**	**<0.0001**	1.27 (0.60, 2.68)	0.52	1.36 (0.63, 2.90)	0.42
	Agree	469	63.0	58	57.4	0.83 (0.52, 1.32)	0.44	0.89 (0.55, 1.41)	0.55	0.87 (0.54, 1.40)	0.58
**Condoms aren’t needed unless you have many partners**	Disagree	585	78.8	80	79.2	1.00	-	1.00	-	1.00	-
	Undecided	25	3.4	3	3.0	0.88 (0.26, 2.97)	0.64	0.82 (0.24, 2.80)	0.75	0.93 (0.27, 3.24)	0.90
	Agree	132	17.8	18	17.8	0.99 (0.58, 1.71)	0.99	0.89 (0.51, 1.54)	0.67	1.01 (0.57, 1.77)	0.98
**There’s pressure to have sex by certain age**	Disagree	288	38.7	25	24.7	1.00	-	1.00	-	1.00	-
	Undecided	76	10.2	4	4.0	0.6 (0.20, 1.79)	0.36	0.58 (0.19, 1.71)	0.32	0.49 (0.16, 1.46)	0.20
	Agree	380	51.1	72	71.3	**2.18 (1.35, 3.52)**	**0.001**	**1.80 (1.09, 2.94)**	**0.02**	1.52 (0.92, 2.51)	0.10
**It’s boring to have the same sex partner for a longtime**	Disagree	490	65.9	50	50.0	**1.00**	**-**	1.00	-	1.00	-
	Undecided	94	12.6	18	18.0	**1.88 (1.04, 3.35)**	**0.03**	1.70 (0.95, 3.06)	0.08	1.32 (0.72, 2.39)	0.37
	Agree	160	21.5	32	32.0	**1.96 (2.21, 3.16)**	**0.01**	1.58 (0.96, 2.58)	0.07	1.43 (0.86, 2.37)	0.16
**Sexual intercourse is an intimate experience**	Disagree	26	3.5	6	5.9	1.00	-	1.00	-	1.00	-
	Undecided	31	4.2	7	6.9	1.00 (0.58, 1.74)	0.99	0.96 (0.55, 1.67)	0.88	1.26 (0.69, 2.30)	0.45
	Agree	690	92.4	88	87.1	0.55 (0.22, 1.38)	0.20	0.60 (0.24, 1.51)	0.28	0.60 (0.23, 1.54)	0.29
**Only two people who trust each other should have sexual relations**	Disagree	195	26.0	46	45.1	1.00	-	1.00	-	1.00	-
	Undecided	93	12.4	19	18.6	**2.92 (2.21, 3.85)**	**<0.0001**	**2.70 (2.03, 3.58)**	**<0.0001**	**2.59 (1.90, 3.53)**	**<0.0001**
	Agree	463	61.7	37	36.3	**0.34 (0.21, 0.54)**	**<0.0001**	**0.41 (0.25, 0.66)**	**<0.0001**	**0.5 (0.30, 0.81)**	**0.01**
**It’s fun to experiment with strangers**	Disagree	371	49.8	29	28.5	1.00	-	1.00	-	1.00	-
	Undecided	121	16.2	19	18.6	**2.01 (1.08, 3.70)**	**0.03**	1.70 (0.90, 3.17)	0.09	1.18 (0.62, 2.23)	0.60
	Agree	253	34.0	54	52.9	**2.73 (1.69, 4.40)**	**<0.0001**	**2.05 (1.21, 3.43)**	**0.01**	1.53 (0.90, 2.58)	0.11
**How often do you think your peers take drugs or alcohol before sex**	A little – never	174	25.1	10	10.1	1.00	-	1.00	-	1.00	-
	Sometimes	369	53.2	45	45.5	**2.12 (1.04, 4.30)**	**0.04**	**2.17 (1.06, 4.41)**	**0.03**	1.96 (0.95, 4.01)	0.07
	A lot	150	21.7	44	44.4	**5.1 (2.48, 10.49)**	**<0.0001**	**5.35 (2.59, 11.05)**	**<0.0001**	**4.76 (2.28, 9.93)**	**<0.0001**

Bolded p-values are statistically significant at critical α=0.05.

^1^Controlling for sex, nationality, current relationship status, living abroad after the age of 12, and spirituality.

## Discussion

Globally, this is the first study to link youths’ consumption of alcohol/drugs at sexual debut with factors such as social pressures and norms, less than safe practices in first-time sexual encounters with unfamiliar partners and increased number of lifetime partners. While these factors have been shown to drive sexual activity, this study highlights their strong relatedness to engaging in first-time sex while under the influence of alcohol and/or drugs.

This study is also the first to examine sexuality and sexual practices among youth from the Arab world, and found that more than 1 in 10 sexually active university youth had consumed alcohol and/or drugs during their first penetrative sexual encounter (i.e. oral, anal or vaginal sex)- a practice more common among males, a non-Arab foreign nationals, students living alone or who have lived abroad for a significant period (especially if in more than one place), as well as the less religious/spiritual.

Unlike in the case of Bellis and colleagues (2008), however, we found no significant differences in age at sexual debut among those who consumed alcohol and/or drugs at the same time versus not. Nonetheless, students who used alcohol/drugs at sexual debut in this sample seem potentially at higher odds of lesser than safe practices during first-time sex and subsequent sexual encounters, as others have found [7,15]. Particularly, they were twice (or more) as likely to engage in first oral or vaginal sex with an unfamiliar partner (i.e. random partner or sex worker), indicative of a low level of intimacy at sexual debut [15] and a higher likelihood of contracting STIs due to an increased odds of non-condom use [29,30]. Using drugs/alcohol at sexual debut was also linked to a higher number of lifetime sexual partners, which constitutes an increased risk of contracting or spreading human immunodeficiency virus (HIV) and other STIs particularly in the absence of safer sex and condom use [31-33]. While they were equally consenting at sexual debut, students who used alcohol/drugs at their first sexual encounter were more likely to admit ‘*ever doing something sexual they did not want to do’*, a matter that has multiple implications at the level of individual autonomy, bodily rights, and public health; worth noting is its persistent strong association after further adjusting for students’ sexual practices, values and perceptions as well as sociodemographics that were independently linked to ‘sexual debut while using alcohol/drugs’ (OR = 2.17; CI = [1.07; 4.37]; p-value = 0.031).

The fact that students who consumed alcohol/drugs at sexual debut were not fearful of doing more sexually than intended due to alcohol/drug use, may possibly be indicative of a strategic consumption of alcohol/drugs to enhance the sexual experience [9], or a lack of sufficient sexual health knowledge about the risks involved, keeping in mind that we do not know the extent to which substances are typically consumed or the decision-making capacity of this sample at sexual debut.

Perceived peer pressure to have sex by a certain age, and peer norm to consume alcohol/drug before sex were correlated strongly with students’ own use of substances at sexual debut, in line with other studies highlighting the pivotal role of peers- whereby perceptions of the prevalence of peers' sexual behaviors was the most important peer normative predictor of intention and initiation of intercourse [34,35]. In our study, students who perceived that their peers took drugs or alcohol before sex ‘a lot’ were more likely to have used alcohol/drug use at their sexual debut, controlling for sexual practices, values and perceptions in addition to sociodemographics that were independently linked to ‘sexual debut while using alcohol/drugs’ (OR = 3.51; CI = [1.43; 8.63]; p-value = 0.006).

Communication patterns did not seem to differentiate youth who engaged in first time sex while using alcohol/drugs from those who did not. This may be partially explained by the fact that the overwhelming majority felt they could not speak to their parents about sex; alternatively the measure may not be precise as it does not characterize the context or the frequency or the details of the sexual discussions with either or both parents. In contrast, and despite that the overwhelming majority felt comfortable talking to their close friends about sex, the sexually active students whose sexual debut happened while using alcohol/drugs were still at a higher odds of discussing sexual topics with their close friends, even when controlling for their discussion with either or both parents. This finding underscores the importance of increasing awareness and knowledge about sexual health among youth in general– as they tend to resort to one another for the most part for such information. This also stresses the need to increase parental knowledge and skills to create more opportunities to communicate with their children- and potentially buffer the effects of negative peer pressure [36]. Despite its contribution to limited existing literature on this topic, particularly from the Arab region, this study is not void of limitations. Our findings are not generalizable to other university students (i.e. public university students) or other youth in Lebanon or the region. Nonetheless, the sample was drawn from one of the largest private universities in Lebanon, and participants were not representative but quite diverse with regards to their socio-demographics, sexual perceptions, behaviors and communication patterns. Temporality could not be ascertained for many of the associations, as in all cross-sectional surveys; still, for some, the nature of the measure inferred temporality (e.g., number of lifetime sexual partners occurred subsequent to sexual debut). While several variables described the context of the first sexual encounter (e.g., extent of willingness to engage in sexual activity, familiarity with partner…), other important data was not collected (e.g., reason or context for substance use, the amount consumed).

## Conclusions

This potential reality of strong perceived peer pressures and norms, limited capacity to communicate with a trusted guardian about sex, and the desire to explore sexually highlights a need to advocate for sexuality education among youth, in schools and at home. Quite important are the strong associations between use of alcohol/drugs at sexual debut and other risky practices that are unlikely to differ among sexually active students of a similar profile. Our findings highlight a strong association between using alcohol and/or drugs at sexual debut (versus not) and various practices that increase youth health risks, including engaging in sexual activity with an unfamiliar partner, having a higher number of lifetime partners, engaging in sexual activity unwillingly. Our study findings also suggest that youth whose first sexual experience took place while using substances may have a different profile of sexual perceptions/values than their also sexually active counterparts. Still, more research is needed to help better understand youth choices at sexual debut, and the reasoning or underlying circumstances behind those choices. Focus group discussions with youth are particularly essential to help contextualize youth choices, and unravel important localized circumstances surrounding their decisions and/or behaviors.

Future studies can also investigate the interplay between sexual activity and substance use, beyond sex debut. Possible directions could include understanding how peer pressures and norms to engage in sexual activity interact with pressure to use alcohol and/or drugs, or even the extent of dependence on substances during sexual interaction. Programs and interventions to address youth sexual practices and substance use have, for the most part, been developed and implemented independently, a reality that may be potentially challenging the success of either or both prevention efforts. Future research that attempts to understand both youth behaviors from a broader public health perspective can provide insight and better chances of preventing youth substance use and enhancing their sexual health wellbeing.

## Abbreviations

STI: Sexually transmitted infection; FHI: First heterosexual intercourse; MSM: Men who have sex with men; IRB: Institutional Review Board; HRPP: Human Research Protection Program; HIV: Human immunodeficiency virus.

## Competing interests

The authors declare that they have no competing interests.

## Authors’ contributions

LG is the principal investigator on the grant and was responsible for the planning and the conduct of all stages of the research study, and write up of this manuscript. FM was involved in the literature review; data analyses and write up of the manuscript. RY was extensively involved in the conceptualization and implementation of the study, and contributed to data interpretation and write up of the manuscript. FEK is the co-principal investigator on the grant and was extensively involved in the conceptualization and implementation of the study, and contributed to data interpretation and write up of the manuscript. All authors read and approved the final manuscript.

## Pre-publication history

The pre-publication history for this paper can be accessed here:

http://www.biomedcentral.com/1471-2458/14/671/prepub
